# Editorial: Innovations in the assessment and treatment of TBI and co-occurring conditions in military connected populations

**DOI:** 10.3389/fneur.2025.1774411

**Published:** 2026-01-14

**Authors:** Jay M. Uomoto, Chandler Sours Rhodes, Jesse R. Fann

**Affiliations:** 1 Henry M. Jackson Foundation for the Advancement of Military Medicine, Inc., in Support of Creative Forces: National Endowment for the Arts Military Healing Arts Network, Bethesda, MD, United States; 2 National Intrepid Center of Excellence (NICoE), Bethesda, MD, United States; 3 Walter Reed National Military Medical Center, Bethesda, MD, United States; 4 Department of Medical and Clinical Psychology, University of the Health Sciences, Bethesda, MD, United States; 5 Department of Diagnostic Radiology and Nuclear Medicine, University of Maryland Medical School, Bethesda, MD, United States; 6 Department of Psychiatry and Behavioral Sciences, University of Washington School of Medicine, Seattle, WA, United States; 7 Department of Rehabilitation Medicine, University of Washington School of Medicine, Seattle, WA, United States

**Keywords:** assessment, military, post-traumatic stress disorder, traumatic brain injury, treatment, veteran

Treatment for the consequences of head injuries dates to the time of Hippocrates (1), when the practice of trephination of the skull was likely considered an innovative treatment. Ganz (2) observes that the evolution of head injury treatment did not occur until the 1700s, when the brain was recognized as the source of symptoms after injury, leading to improvements in neurosurgical interventions. In the United States, engagement in wars from the Civil War to recent conflicts has prompted the advancement of assessment and rehabilitation strategies (3), which is the core theme of this special topic.

This Research Topic highlights innovations and new developments in the assessment, treatment, and neurorehabilitation of military service members and veterans with traumatic brain injury (TBI) and common co-occurring disorders. The goal is to stimulate discussion and further research into effective means of enhancing the quality of life and healthcare outcomes in military-connected populations. Our special topics issue represents articles in the areas of family wellness, biomarkers, interventions, and creative arts therapy.

## Family wellness

While rehabilitation efforts following TBI have historically centered around interventions focused on the patient, increasing attention has shifted to include research into the impact of social support and family functioning on long-term rehabilitation outcomes for service members following injury. This shift in focus is highlighted within this special topic through a set of manuscripts (Brickell, Wright, et al.) describing the work of the Caregiver and Family Member Study of the United States Defense and Veterans Brain Injury Center-Traumatic Brain Injury Center of Excellence (DVBIC-TBICoE) 15-Year Longitudinal TBI Study. Building upon their previous work, Brickell, French, et al. provide evidence that following TBI, service members living in unhealthy family environments report worse health-related quality of life outcomes compared to those living in healthy family environments. Furthermore, using a dyadic approach (Brickell, Ivins, et al.) they show that both service members and intimate partners who are dissatisfied in their relationship report worse health outcomes compared to their satisfied counterparts. Finally, they describe their work to translate their research findings into a clinical Family Wellness Program that is in the initial stages of implementation at the National Intrepid Center of Excellence and planned expansion across the Defense Intrepid Network for TBI and Brain Health. This work paves the way for “[f]amily wellness to become a long-term component of DoD TBI treatment programs, promoting a holistic, family-centered interdisciplinary model of care that supports service member brain health, return to duty following a TBI, and the development of healthy, resilient, and mission-ready military families.”

## Biomarkers

Significant work has been conducted in recent years to investigate biomarkers associated with TBI and comorbid conditions. The current special topic includes a systematic review by Cowansage et al. who evaluated genetic and peripheral biomarkers related to TBI and post-traumatic stress disorder. In a study by Shura et al., olfactory identification performance was hypothesized to distinguish the effects of TBI from post-traumatic stress disorder (PTSD) in post-deployed service members and veterans. Using the University of Pennsylvania Smell Identification Test, they found that individuals with a history of moderate/severe TBI showed reduced olfactory abilities. In contrast, PTSD symptoms and symptom clusters did not meaningfully relate to olfactory performance. Overall, the study suggests that reduced smell identification is linked to TBI but not PTSD, indicating potential clinical utility for assessing TBI-related impairment.

## Interventions

There continues to be a great need for cost-effective intervention options for the treatment of TBI and co-occurring conditions in military-connected populations. This issue includes several novel approaches in varying degrees of development. Fann et al. describe an analysis of a telehealth intervention that was built on a problem-solving framework and used brief modules tailored to target specific common co-occurring conditions among active-duty service members with a history of mild TBI. This approach was found to be feasible and acceptable to service members while being effective in decreasing symptoms. Specifically, they report that the overall number of significant comorbidities and symptoms of PTSD, depression, anxiety, and sleep were significantly improved from baseline to 6 months, compared to an education control. While encouraging, improvements were not sustained at 12 months, suggesting that maintenance strategies need to be further developed and tested. In a series of retrospective chart reviews, Frueh et al. report that alpha-guided repetitive transcranial magnetic stimulation (alpha-rTMS) was beneficial and well-tolerated in reducing post-concussive and PTSD symptoms in special operators with comorbid TBI and PTSD.

It is well-documented that treatment effectiveness and adherence with symptom reporting are common challenges among service members and veterans with TBI. These issues are particularly problematic in the active-duty setting, where return to duty is a priority. Monti et al. showed that microinteraction ecological momentary assessment (mEMA) to collect symptom and treatment engagement information delivered via smartwatch is feasible and acceptable among service members with a history of TBI, including those undergoing cognitive rehabilitation for cognitive impairment. In a randomized controlled trial comparing a novel advanced reasoning training intervention (SMART) to a traditional cognitive rehabilitation program in service members with mild TBI, Darr et al. found that SMART was similarly effective and more cost- and time-efficient in reducing cognitive deficits and post-concussive symptoms. By using meta-cognitive strategies that focus on advanced reasoning skills, SMART offers an alternative to traditional compensatory-based approaches. Dieterich-Hartwell et al.'s detailed case report in this issue highlights the benefit and potential rapid improvement in symptoms from an intensive, tailored, outpatient interdisciplinary team approach delivered to patients with TBI and a complex constellation of comorbidities.

## Creative arts therapy

The field of creative arts therapies is emerging as a potentially effective intervention in military-connected populations with TBI and co-occurring conditions. These therapies are generally well-accepted by patients with a favorable safety profile, thus being a good fit for this population with cognitive and behavioral health co-occurring symptoms.

Chilton et al. present a retrospective case study showing that culturally informed art therapy helped an African American female combat veteran with mild TBI and PTSD express emotions, communicate needs, and explore cultural identity through thematic analysis and standardized emotion-regulation measures. Introduced by Vetro-Kalseth et al., the Auditory Cognition Lab is a collaborative music therapy and speech-language pathology program designed for military-connected individuals with TBI, demonstrating preliminary improvements in auditory processing, cognition, language, attention, and self-efficacy, while outlining a framework for future feasibility research. Story et al. examine veterans' experiences in the Feasibility and Acceptability of Music Imagery and Listening Interventions for Analgesia (FAMILIA) project, a telehealth-delivered music-listening and music imagery intervention for chronic pain, finding that participants viewed both approaches as acceptable, motivating, and beneficial for managing pain and psychological symptoms despite some logistical challenges. Finally, Gooding et al. evaluate a community-based music therapy program delivered in supportive housing for veterans experiencing homelessness, TBI, and co-occurring conditions. Their findings identify preferred music styles, common intervention strategies, and themes of self-expression, rapport, and group cohesion, with results indicating that onsite, collaborative music programming can foster trust, connection, and improved wellbeing among this marginalized population.

## Summary and future innovations

Many promising avenues for evaluating and treating individuals with TBI and co-occurring conditions such as PTSD have yet to be fully explored. Emerging assessment technologies are likely to integrate self-report, clinician ratings, physiological monitoring, and validated neuroimaging and blood biomarkers to create a more complete and dynamic picture of military service members and veterans with complex post-TBI presentations. Developing this holistic, data-rich understanding of each patient will help bridge the gap between assessment and targeted intervention, advancing us toward truly personalized and precision-based care.

Military service is associated with additional occupational risk factors that impact both short- and long-term health outcomes, such as repetitive low-level blast exposure, extreme psychological and physiological stress and trauma, chemical exposures, disrupted sleep patterns, as well as the impacts of training and deployment cycles on social relationships. The recently introduced concept of the exposome [see ref. (4)], which is used to describe how internal and external environmental exposures experienced throughout life impact health, is especially relevant to this population and the conceptualization of TBI as a chronic health condition (5). Therefore, future research in this area requires an integrated approach to incorporate the individual and combined impacts of TBI and military-specific occupational exposures in the development and evaluation of targeted interventions.

A forward-looking research agenda for military service members and veterans must extend beyond improving proximal clinical outcomes alone. To be truly impactful, innovative assessment and treatment strategies should explicitly connect near-term clinical gains to the distal outcomes that matter most for each population. For active-duty service members, this means designing and evaluating interventions that not only reduce symptoms but also strengthen resilience and military readiness. For veterans, research must link clinical improvements to meaningful participation in everyday activities and life roles, as articulated within the International Classification of Functioning (ICF). By anchoring proximal outcomes to these longer-term, population-specific goals, future research can better support recovery, reintegration, and sustained wellbeing.

## Author disclaimer

The opinions contained herein represent the private views of the authors and are not to be construed as official or as reflecting the views, opinions, or policies of the Henry M. Jackson Foundation for the Advancement of Military Medicine, Inc., the National Endowment for the Arts, Department of War, or the U.S. Government. Mention of trade names, commercial products, or organizations does not imply endorsement by the U.S. Government. This material was created free of branding or market affiliations. The authors are operating solely as contributors.
